# Cognitive training with and without additional physical activity in healthy older adults: cognitive effects, neurobiological mechanisms, and prediction of training success

**DOI:** 10.3389/fnagi.2015.00187

**Published:** 2015-10-13

**Authors:** Julia Rahe, Jutta Becker, Gereon R. Fink, Josef Kessler, Juraj Kukolja, Andreas Rahn, Jan B. Rosen, Florian Szabados, Brunhilde Wirth, Elke Kalbe

**Affiliations:** ^1^Center for Neuropsychological Diagnostics and Intervention (CeNDI), Institute of Gerontology, University of Vechta Vechta, Germany; ^2^Department of Neurology, University Hospital Cologne Cologne, Germany; ^3^Institute of Human Genetics, University Hospital Cologne Cologne, Germany; ^4^Cognitive Neuroscience, Institute of Neuroscience and Medicine (INM-3), Research Center Jülich Jülich, Germany; ^5^Department of Geriatrics, St. Franziskus Hospital Lohne Lohne, Germany; ^6^Laboratory Services Laborarztpraxis Osnabrück Osnabrück, Germany; ^7^Department of Medical Psychology, University Hospital Cologne Cologne, Germany

**Keywords:** combined lifestyle intervention, predictor, moderator, neurobiological mechanisms, motivation

## Abstract

Data is inconsistent concerning the question whether cognitive-physical training (CPT) yields stronger cognitive gains than cognitive training (CT). Effects of additional counseling, neurobiological mechanisms, and predictors have scarcely been studied. Healthy older adults were trained with CT (*n* = 20), CPT (*n* = 25), or CPT with counseling (CPT+C; *n* = 23). Cognition, physical fitness, BDNF, IGF-1, and VEGF were assessed at pre- and post-test. No interaction effects were found except for one effect showing that CPT+C led to stronger gains in verbal fluency than CPT (*p* = 0.03). However, this superiority could not be assigned to additional physical training gains. Low baseline cognitive performance and BDNF, not carrying apoE4, gains in physical fitness and the moderation of gains in physical fitness × gains in BDNF predicted training success. Although all types of interventions seem successful to enhance cognition, our data do not support the hypotheses that CPT shows superior CT gains compared to CT or that CPT+C adds merit to CPT. However, as CPT leads to additional gains in physical fitness which in turn is known to have positive impact on cognition in the long-term, CPT seems more beneficial. Training success can partly be predicted by neuropsychological, neurobiological, and genetic parameters. Unique Identifier: WHO ICTRP (http://www.who.int/ictrp); ID: DRKS00005194.

## Introduction

Combined interventions that target at multiple factors, e.g., the combination of cognitive training (CT) with physical activity (CPT), have recently been suggested to be most effective in maintaining or even improving cognitive health (Bamidis et al., 2014; Law et al., 2014). First controlled trials indeed found that CPT lead to superior improvements in different cognitive domains (Fabre et al., 2002; Oswald et al., 2006; Theil et al., 2013; Rahe et al., 2015b). In contrast, recent randomized controlled trials (RCT) failed to observe superior effects of CPT (Legault et al., 2011; Barnes et al., 2013; Shatil, 2013; Linde and Alfermann, 2014). Thus, data is inconsistent—probably partly due to the heterogeneity of studies with regard to interventions and study designs—and data is too rare to draw clear conclusions. In addition, it should be noted that most CPT studies did not include multi-domain cognitive (Fabre et al., 2002; Oswald et al., 2006; Legault et al., 2011; Barnes et al., 2013; Theil et al., 2013) and multi-component physical activity (Fabre et al., 2002; Oswald et al., 2006; Legault et al., 2011; Barnes et al., 2013; Shatil, 2013; Theil et al., 2013; Linde and Alfermann, 2014) and thus neglect recent recommendations for efficient interventions of cognitive aging (for physical training see Tseng et al., 2011; for CT see Cheng et al., 2012; Rebok et al., 2014). Furthermore, participants' motivation is a crucial factor that strongly influences whether a cognitive or physical intervention is effective (see Carretti et al., 2011; Lautenschlager et al., 2012). In line with this, a recent controlled trial with follow-up assessment indicated that the motivation to remain physically active after the training itself seems to mainly drive superior effects of CPT vs. CT (Rahe et al., 2015b). CPT involving motivational physical activity counseling has not been studied yet.

Importantly, to date the biological mechanisms of the cognitive effects of CPT remain elusive. Both cognitive (Mozolic et al., 2010; May, 2011; Chapman et al., 2013) and physical activity (Erickson et al., 2012b; Carvalho et al., 2014) have been shown to induce neuronal and cognitive plasticity in the aging brain. According to this notion, stronger benefits can be expected for the use of additive or synergistic effects of CPT (Curlik II and Shors, 2013). In line with this, it has been suggested that physical activity might improve brain metabolism and plasticity directly and that an ensuing cognitive activity might use the enhanced brain metabolism and guide the plasticity processes resulting in stronger cognitive improvement after CPT (Oswald et al., 2006; Bamidis et al., 2014). Note, however, that the latter hypothesis is based on animal research (Curlik II and Shors, 2013) and that the effects of CPT upon the processes underlying neuronal and cognitive plasticity in humans remain to be elucidated.

Animal and human research on biological mechanisms provoked by lifestyle activities mutually have focused on neurotropic growth factors that seem to play a crucial role in brain plasticity (for review see Valenzuela et al., 2007; Lista and Sorrentino, 2010). Especially brain-derived neurotropic factor (BDNF), insulin-like growth factor 1 (IGF-1), and vascular endothelial growth factor (VEGF) have been studied as complementary indicators of exercise induced neuro-, synapto-, and angio-genesis (for an overview see Cotman et al., 2007; Erickson et al., 2012b; Coelho et al., 2013; Voss et al., 2013b). Recently, Voss et al. (2013a) found that increased peripheral BDNF, IGF-1, and VEGF after an aerobic walking intervention were associated with improved connectivity between temporal lobe structures. As no cognitive changes were assessed in that study, the association of changes in growth factors with cognitive outcomes needs investigation (Voss et al., 2013a). Furthermore, to our knowledge, effects of combined lifestyle interventions on growth factors have not been reported yet. Therefore, further research is mandatory to strengthen the assumption—that combined interventions are superior—with evidence supporting complementary biological mechanisms.

Several variables may have predictive value for cognitive improvement resulting from non-pharmacological interventions, such as cognitive baseline performance (Fairchild et al., 2012; Whitlock et al., 2012), education (Olazarán et al., 2004), age (Aguirre et al., 2012), APOE polymorphism (Binetti et al., 2013), BDNF polymorphism (Erickson et al., 2012a), sex (Aguirre et al., 2012), and baseline peripheral growth factors (Voss et al., 2013a). Yet again, considerable inconsistencies have been reported, e.g., both high (Fairchild et al., 2012) and low baseline performance (Whitlock et al., 2012) have been associated with CT gains. To the best of our knowledge, predictors of cognitive gains after CPT have not yet been reported.

Accordingly, the main aim of this RCT was to investigate whether CPT leads to stronger cognitive effects than CT. Additionally, effects on physical fitness and the neurotrophic growth factors brain-derived neurotrophic factor (BDNF), IGF-1, and VEGF were assessed and compared between groups. Based on the literature discussed above, the hypotheses of this study are: (1) The effects of CPT on various cognitive domains, physical fitness and peripheral growth factors are superior to that of pure CT in healthy older adults, (2) The effects of CPT with counseling on various cognitive domains, physical fitness and peripheral growth factors are superior to that of CPT without counseling in healthy older adults. In an explorative attempt we finally investigated predictors of cognitive improvement within the CPT intervention group.

## Materials and methods

This study was registered at the WHO ICTRP (ID: DRKS00005194) and conducted as a RCT to investigate whether the effects of CPT on (i) various cognitive domains (primary outcomes) and (ii) physical fitness and peripheral growth factors (secondary outcomes) are superior to that of pure CT in healthy older adults (hypothesis 1). In addition, CPT was contrasted to CPT with motivational physical activity counseling. Here, we expected CPT with counseling to further enhance the effects of CPT on the primary and secondary outcomes (hypothesis 2). Furthermore, predictors of cognitive gains of CPT were analyzed.

### Participants and procedure

The study was approved by the ethics committee of the University Hospital Cologne, Germany, and the medical association of Lower Saxony, Germany. Healthy older adults were recruited in Vechta, Osnabrück, and Cologne, Germany. Interested subjects were interviewed about their personal data and history of diseases by phone. All participants gave written informed consent in accordance with the Declaration of Helsinki prior to the first neuropsychological assessment and in case of cardiovascular disease affirmation of the participants' general practitioner re principal eligibility for this study was obtained. Participants were invited to a first screening appointment in which eligibility was further evaluated; a detailed medical history was obtained, age-adequate cognitive state was verified with the cognitive screening DemTect (Kalbe et al., 2004), and depressive symptoms were assessed with the German Beck Depression Inventory 2 (BDI2; Hautzinger et al., 2009). Inclusion criteria were as follows: age 50–85 years, normal or corrected-to-normal vision and hearing, and German native speaker. Exclusion criteria were: any psychiatric or neurological disease (past or present), a condition that prohibited moderate physical activity, past or present intake of psychotropic drugs, cognitive state below the normal range in the DemTect (≤ 12 points; Kalbe et al., 2004), presence of clinically relevant symptoms of depression as assessed with the BDI2 (≥20 points; Hautzinger et al., 2009), and former participation in a cognitive group training. Participants who attended < 80% of the training sessions were subsequently excluded from the study.

We used the online Research Randomizer (http://www.randomizer.org) to randomize participants to interventions. Due to the study design with an intended number of subjects of *N* = 90 participants in three study centers (Vechta, Osnabrück, and Cologne, Germany), we planned three training groups with a total of *n* = 30 participants per study center. After the screening, randomization in blocks of three was conducted for the allocation of participants as well as intervention types to one of the three groups, separately for each study center. Participants were not blinded for training groups and were told that the aim of the study was to compare different interventions.

### Interventions

Participants were trained with one of three interventions for 7 weeks by a certified trainer. In order to investigate time- and cost-efficient interventions of cognitive decline, training programs with rather short duration of 7 weeks were chosen intentionally. Cognitive effects of CPT have already been reported after training programs of 7 to 8 weeks (Schneider and Yvon, 2013; Rahe et al., 2015b). Training sessions with a maximum of 10 participants per group were carried out twice weekly with a total of 14 sessions each lasting 90 min in all three interventions. The three interventions were based on standardized manuals and all of them contained the multi-domain CT NEURO*vitalis* (Baller et al., 2009; Petrelli et al., 2014; Rahe et al., 2015a,b). For CPT interventions, CT was supplemented with a multi-component physical activity program and two additional sessions about *Physical Activity* and *Nutrition*. The CPT intervention with physical activity counseling included additional motivational counseling in the first and the last training week. Training amount was comparable between the three intervention groups. A detailed description of the interventions and their comparison is given in the Supplementary Material.

### Outcome measures

Primary and secondary outcomes were assessed at pretests and posttests in standardized test situations. Assessors had been trained intensely in test application and scoring and were blinded for participants' training group allocation. Primary outcomes of the study were performance changes in the domains of memory (Kalbe et al., 2004; Strauss et al., 2006), attention (Strauss et al., 2006), executive functions (Bäumler, 1985; Wilson et al., 1996; Aschenbrenner et al., 2000; Kalbe et al., 2004; Aster et al., 2009), visuo-construction (Strauss et al., 2006), and general cognitive state (Kalbe et al., 2004; see **Table 2** for the cognitive measures). If available, parallel forms were used and use of versions A/B in pre- and post-tests were randomized to minimize retest effects. Secondary outcomes were changes in physical fitness assessed with the Senior Fitness Test (Rikli and Jones, 2001; see **Table 3** for the physical measures) which provides reliable and valid measurement of all targeted components of the physical activity program (strength, endurance, flexibility, coordination). Peripheral blood levels of BDNF, IGF-1, and VEGF were assessed at pre- and post-test. Depending on the study center, blood sampling and preanalytics were performed at Central Laboratory, St Franziskus Hospital Lohne (Vechta), at Laboratory Dr. Enzenauer and Associates (Osnabrück), or at the Central Laboratory, University Hospital Cologne (Cologne). For the assessment of IGF-1, 4.7 ml blood was collected in a Serum-gel Monovette® with clotting-activator (Sarstedt, Germany) and was kept at 6–8°C for 30 min to allow for clotting. Afterwards samples were centrifuged at 3399g at 4°C for 30 min. Serum samples were frozen at −34°C and transported to the Central Laboratory Cologne, where peripheral IGF-1 was identified within 1 week after arrival of the samples with Chemilumineszenz-Immunoassay (Siemens, Germany, until December 2012, afterwards this was not deliverable anymore), respectively Sandwich-Chemilumineszenz-Immunoassay (DiaSorin, Italy, from January 2013). Equality of values was verified with a comparison of both assays (W. Hein, personal communication, January 2013). For the assessment of VEGF and BDNF, 9 ml blood was collected in an EDTA Monovette® (Sarstedt, Germany), cooled in ice water, and within 20 min after withdrawal centrifuged at 3399g at 4°C for 30 min. The separated plasma was centrifuged at 3399g at 4°C for another 20 min to remove thrombocytes completely. Aliquoted plasma was frozen at −34°C and transported to the MVZ Laboratory Dr. Eberhard and Partner in Dortmund, Germany. Aliquotes of all participants from both pre- and post-tests were defrosted in November 2013 and Quantikine ELISA Human BDNF (R&D Systems, Germany) was used for the estimation of peripheral BDNF and VEGF-A ELISA (IBL international, Germany) was used to estimate peripheral VEGF. Quantification of growth factor levels was performed following manufacturer's instructions. For genotyping of the APOE and BDNF polymorphisms, 400 μl EDTA blood was used for automated DNA isolation with the *Maxwell*® 16 System, applying the *Maxwell*® 16 Blood DNA kit according to the manufacturer's protocol (Promega, Madison/Wisconsin, USA). For APOE-genotyping, the polymorphisms rs7412: (c.526C>T; p.Arg176Cys) and rs429358 (c.388T>C; p.Cys130Arg) were determined. Therefore, we performed directed PCR amplification and Sanger DNA sequencing of the variant loci (*APOE4* gene; OMIM 107741; RefSeq NM_000041.2; exon 4). Samples were classified for both codons either as homozygotes (either arginine or cysteine on both alleles) or as heterozygotes (each arginine and cysteine on one allele). Presence of an apoE-2-allele (p.130Cys, p.176Cys), an apoE-3-allele (p.130Cys, p.176Arg), and an apoE-4- allele (p.130Arg, p.176Arg) was assessed by the combination of the genotypes of the two SNPs. Furthermore, the BDNF variation rs6265 (c.196G>A; p.Val66Met) was analyzed. We performed direct PCR amplification and Sanger DNA sequencing of the region of interest (*BDNF* gene; OMIM 113505; RefSeq NM_001143807.1; exon 2). Sequence data were analyzed automatically using the program SeqPilot (JSI GmbH; Medical Systems, Germany).

### Statistical analyses

IBM SPSS Statistics 21 for Windows (2013) was used for data analyses. Normal distribution of data was tested with Kolmogorov-Smirnov tests and homogeneity of variances for between-group comparisons was tested with Levene's tests. Baseline demographics were compared between groups as well as between participants and drop-outs using ANOVAs, Kruskal-Wallis tests, or Chi-square tests. The significance level of all contrasts was set at α = 0.05. *Post-hoc* power analyses were performed using G^*^Power (Faul et al., 2009) to estimate achieved power.

According to our hypotheses, we treated the study as two separate trials comparing alternative interventions with trial 1 comparing CPT vs. CT (hypothesis 1) and trial 2 comparing CPT vs. CPT+C (hypothesis 2) in face-to-face comparisons. Changes from pre- to post-test were analyzed with ANOVAs for repeated measures (rANOVAs). The within-subject variable *Time* had two levels (pre- vs. post-test). The between-subject variable *Training* had two levels (trial 1: CPT vs. CT, trial 2: CPT vs. CPT+C). To handle potential violations of sphericity, Greenhouse-Geisser values are reported. We report the effect size partial η^2^ (η^2^_p_) indicating a small (η^2^_p_ > 0.01), moderate (η^2^_p_ > 0.06), or strong effect (η^2^_p_ > 0.14; Field, 2009). Pairwise comparisons with Bonferroni correction were calculated for the significant *Time* effects of the rANOVAs with an overall α = 0.05. For these analyses we report the effect size *d* indicating a small (*d*>0.10), moderate (*d*>0.30), or strong effect (*d*>0.50; Field, 2009). If the assumptions of normal distribution and homogeneity of variances were violated or variables were non-parametric in nature, Friedman's ANOVA was used and the effect size ω is reported indicating a small (ω > 0.10), moderate (ω > 0.30), or strong effect (ω > 0.50; Field, 2009). To compare the training gains within the groups, Wilcoxon signed-rank test was used as a *post-hoc* test for the significant variables of Friedman's ANOVAs (Field, 2009). The Bonferroni procedure was applied manually for non-parametric *post-hoc* tests to prevent an inflated type I error (0.05/number of comparisons). We report the effect size ϕ indicating a small (ϕ > 0.10), moderate (ϕ > 0.30), or strong effect (ϕ > 0.50; Field, 2009) for the Wilcoxon signed-rank tests. To estimate between-group differences at the two assessments the non-parametric Kruskal-Wallis test was used and ϕ is reported as effect size.

Predictions of cognitive improvement were exclusively calculated for CPT, because predictors of combined interventions have not been investigated so far. Predictors of cognitive improvements within CPT were estimated using backwards multiple regressions. We calculated the change scores (Δ = post − pre) of cognitive variables, peripheral growth factors, and overall physical fitness. Based on the current literature, the predictors age, education, sex, APOE, and BDNF polymorphisms, baseline cognitive scores, baseline levels of BDNF, IGF-1, and VEGF, baseline overall physical fitness, change in growth factors, and change in overall physical fitness were integrated in the regression models. To analyze the possible relationship between gains in overall physical fitness and gains in peripheral growth factors, we considered interactions between *changes in physical fitness* × *changes in peripheral growth factors* for the analysis of possible moderator effects (see Aiken and West, 1991) when both the gains in peripheral growth factor and in overall physical fitness were significant predictors. For such moderator analyses, we centered the independent variables and integrated the interaction term of the centered variables as an additional predictor in the model. The assumptions of multiple regression were checked according the suggestions of Field (2009).

## Results

### Participants' flow and baseline characteristics

**Figure 1:**
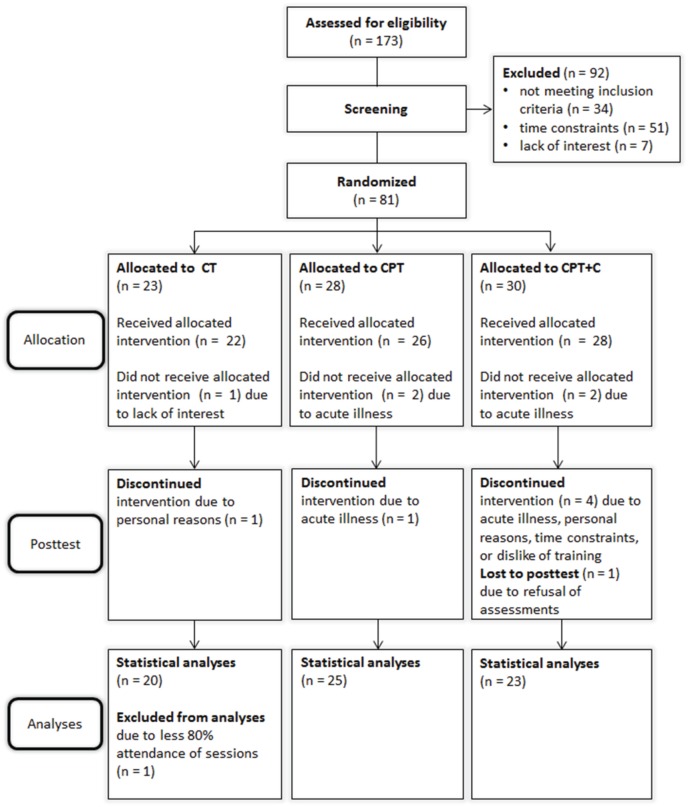
**Flow of participants through the study**. CPT, cognitive-physical training; CPT+C, cognitive-physical training with counseling; CT: cognitive training.

*N* = 173 participants were recruited and assessed for eligibility from October 2012 until June 2013 of which *n* = 81 were randomized. Due to low recruitment numbers in Osnabrück, only two groups were trained at this study center and a third group was trained in Vechta to ensure an appropriate sample size. A total of *n* = 13 participants dropped out during the study. Figure 1 shows the participants' flow through the study. Drop-outs and participants did not differ significantly in age, education, depressive symptoms, cognitive state, or distributions of sex, apoE4-carrier, or BDNF polymorphism (all *p*>0.05).

At pretest, participants of the interventions did not differ significantly in baseline demographics (all *p*>0.05, see Table 1). Due to the small sample size, we categorized participants as apoE-4 carriers (*n* = 16, apoE-2/E-4 or apoE-3/E-4 heterozygotes) vs. apoE-4 non-carriers (*n* = 52) for statistical analyses. In detail, *n* = 2 participants were apoE-2 homozygotes, *n* = 13 were apoE-2/E-3 heterozygotes, *n* = 3 were apoE-2/E-4 heterozygotes, *n* = 37 were apoE-3 homozygotes, and *n* = 13 were apoE-3/E-4 heterozygotes. Distribution of genotypes did not differ between groups, χ^2^_(8)_ = 6.57, *p* = 0.58.

**Table 1:** **Baseline characteristics of the study sample**.

**Demographics**	**CT (*****n*** = 20**)**			**CPT (*****n*** = 25**)**			**CPT**+**C (*****n*** = 23**)**			***p***
	***M***	**(*SD*)**	**Range**	***M***	**(*SD*)**	**Range**	***M***	**(*SD*)**	**Range**	
Age	67.65	(6.86)	53–82	68.44	(7.36)	51–81	69.09	(7.20)	50–85	0.81^a^
Education	14.80	(2.82)	11–20	14.44	(3.34)	11–22	14.22	(3.58)	11–21	0.61^b^
Cognitive status	16.60	(1.70)	13–18	16.52	(1.81)	13–18	16.96	(1.69)	13–18	0.66^a^
Overall physical fitness in %	55.30	(20.07)	9–90	63.11	(15.54)	34–91	63.74	(20.15)	26–94	0.27^a^
Handedness	Right:19	Left: 0	Mixed: 1	Right: 21	Left: 1	Mixed: 3	Right: 21	Left: 0	Mixed: 2	0.65^c^
apoE genotype	E4-Carrier: 2			E4-Carrier: 9			E4-Carrier: 5			0.14^c^
BDNF genotype	Val66Met: 7			Val66Met: 13			Val66Met: 10			0.52^c^
Sex	♀ = 12 ♂ = 8			♀ = 16 ♂ = 9			♀ = 18 ♂ = 5			0.39^c^
	60% 40%			64% 36%			78% 22%			

*Range of DemTect norms for normal cognitive status: 13–18. apoE, Apolipoprotein E; BDNF, brain-derived neurotropic factor; CT, Cognitive training; CPT, Cognitive training with additional physical activity; CPT+C, Cognitive training with additional physical activity and counseling*.

a *Comparison of groups at baseline with ANOVAs*.

b *Comparison of groups at baseline with Kruskal-Wallis test*.

c *Comparison of groups at baseline with Chi-square tests*.

### Group differences in outcome measures

Performances of the intervention groups at pre- and post-test are shown in Tables 2, 3. We achieved a 26% power to detect small interaction effects (η^2^_p_ > 0.01), 91% power to detect moderate interaction effects (η^2^_p_ > 0.06), and 99% power to detect strong interaction effects (η^2^_p_ > 0.14) in the comparisons of the groups for testing hypothesis 1 (*N* = 45; 2-tailed α = 0.05). For testing hypothesis 2, a 27% power to detect small interaction effects (η^2^_p_ > 0.01), 93% power to detect moderate interaction effects (η^2^_p_ > 0.06), and 99% power to detect strong interaction effects (η^2^_p_ > 0.14) in the comparisons of the groups was achieved (*N* = 48; 2-tailed α = 0.05).

**Table 2:** **Primary outcomes of the training groups at pre- and post-test**.

**Domain**	**Max**.	**CT (/n = 20)**		***p***	**CPT (*****n*** = 25**)**		***p***	**CPT/+C (/n = 23)**		***p***
		*****M*** (***SD***) or ***Mdn*** (Range)**			***M*** **(*****SD*****) or** ***Mdn*** **(Range)**			*****M*** (***SD***) or ***Mdn*** (Range)**		
		**Pretest**	**Posttest**		**Pretest**	**Posttest**		**Pretest**	**Posttest**	
**MEMORY**										
**Verbal memory**										
DemTect, *IR*^a^	20	14.10 (2.25)	14.45 (2.33)		14.16 (2.15)	15.32 (2.02)	^*^ ^d^	14.00 (2.56)	14.78 (2.68)	
DemTect, *DR*^a^	10	6.10 (2.92)	6.75 (2.36)		5.76 (2.20)	6.16 (2.38)		6.43 (2.17)	6.04 (2.33)	
**Figural memory**										
CFT, *DR*	1	21.40 (6.00)	24.80 (4.71)	^***^	19.84 (5.56)	23.64 (4.56)	^***^^c^^,^ ^d^	19.74 (5.10)	23.17 (6.60)	^***^
**ATTENTION**										
BTA	20	17.30 (2.68)	17.40 (2.14)		16.08 (2.38)	17.32 (2.10)	^*^ ^d^	16.91 (2.80)	17.57 (1.75)	
**EXECUTIVE FUNCTIONS**										
**Working memory**										
WAIS-II, *DSB*	14	6.80 (2.02)	7.41 (2.11)		6.68 (2.46)	7.68 (2.56)	^*^ ^c^	7.00 (1.81)	7.43 (2.21)	
**Verbal fluency**										
RWT, *total*	–	45.45 (12.17)	49.45 (11.19)	^*^	43.64 (14.00)	47.52 (10.64)	^*^^c^^,^ ^d^	43.87 (11.98)	50.35 (12.89)	^***^
RWT, *G-R %*	90%	52.10 (30.88)	60.40 (31.04)		63.32 (29.48)	67.32 (29.75)		51.39 (35.11)	76.22 (23.38)	
DemTect, *S/A*^a^	4	4.00 (2–4)	4.00 (4)		4.00 (4)	4.00 (4)		4.00 (4)	4.00 (4)	
**Inhibition**										
Stroop Diff.^b^	–	48.60 (17.81)	45.41 (16.62)		46.81 (29.62)	43.89 (17.66)		52.74 (32.83)	47.85 (26.90)	
**Planning**										
Key search^a^	16	14.00 (8–16)	15.00 (6–16)		11.00 (4–16)	12.00 (7–15)		14.00 (8–16)	15.00 (10–16)	^**^
**COGNITIVE STATUS**										
DemTect	18	16.60 (1.70)	17.30 (1.34)		16.52 (1.81)	17.04 (1.54)		16.96 (1.69)	17.00 (1.56)	
**VISUO-CONSTRUCTION**										
CFT, *Copy*^a^	36	35.00 (30–36)	35.00 (30–36)		35.00 (29–36)	34.00 (31–36)		34.00 (26–36)	35.00 (33–36)	

*The DemTect is from Kalbe et al. (2004). The Complex Figure Test (CFT) and the Brief Test of Attention (BTA) as described in Strauss et al. (2006) was used. The German Wechsler Adult Intelligence Scale (WAIS-II) is from Aster et al. (2009). The trials S, P, M (total) and alternating G-R of the Regensburger Wort Flüssigkeits-Test (RWT) from Aschenbrenner et al. (2000) were used. The Stroop Test is from Bäumler (1985). The Key Search is from Wilson et al. (1996). The p-values are shown for the post-hoc pairwise comparisons. CT, Cognitive training; CPT, Cognitive training with additional physical activity; CPT+C, Cognitive training with additional physical activity and counseling; DR, subtest delayed recall; DSB, subtest digit span backwards; G-R %, percentile rank for subtest G-R; IR, subtest immediate recall; η^2^_p_, partial η^2^; NT, subtest number transcoding; S/A, subtest supermarket/animal. Stroop Diff. = [trial 3-M (trial 1 + trial. 2)] (see Van Hooren et al., 2007)*.

a *Medians and Ranges are only displayed for the variables that had to be analyzed with non-parametric methods*.

b *Smaller scores indicate better performance*.

c *p-values of comparison 1 (CPT vs. CT)*.

d *p-values of comparison 2 (CPT vs. CPT+C)*.

* *p ≤ 0.05;*

** *p ≤ 0.01;*

*** *p ≤ 0.001*.

**Table 3:** **Secondary outcomes of the training groups at pre- and post-test**.

**Domain**	**Max**.	**CT (/n = 20)**		***p***	**CPT (*****n*** = 25**)**		***p***	**CPT/+C (/n = 23)**		***p***
		*****M*** (***SD***) or ***Mdn*** (Range)**			***M*** **(*****SD*****) or** ***Mdn*** **(Range)**			*****M*** (***SD***) or ***Mdn*** (Range)**		
		**Pretest**	**Posttest**		**Pretest**	**Posttest**		**Pretest**	**Posttest**	
**PHYSICAL FITNESS**										
Overall	95%	55.30 (20.07)	62.04 (18.30)	^**^	63.11 (15.54)	76.43 (10.86)	^***^^b^^,^ ^c^	63.74 (20.15)	75.02 (16.22)	^***^
**Strength**										
Arm-Curl^a^	95%	75.00 (15–95)	75.00 (10–95)		85.00 (25–95)	95.00 (35–95)	^***^^b^^,^ ^c^	80.00 (25–95)	95.00 (40–95)	^***^
30 S Chair stand^a^	95%	60.00 (0–95)	65.00 (0–95)	^*^	60.00 (15–95)	80.00 (50–95)	^***^^b^^,^ ^c^	70.00 (30–95)	90.00 (30–95)	^***^
**Flexibility**										
Chair sit and reach	95%	31.25 (34.06)	39.41 (34.04)		46.17 (39.54)	63.33 (38.59)	^***^^b^^,^ ^c^	49.13 (40.61)	59.22 (37.65)	
**Coordination**										
8 foot up and go	95%	69.75 (22.27)	73.37 (18.76)		71.00 (11.82)	80.52 (9.57)	^***^^b^^,^ ^c^	69.35 (18.79)	78.67 (11.07)	^***^
**Endurance**										
6 Min walk test/2 Min step test	95%	53.75 (24.65)	58.00 (20.74)		57.40 (26.15)	67.54 (23.26)	^*^ ^b^	60.43 (28.36)	65.87 (29.72)	
**GROWTH FACTORS**										
sqrtBDNF [pg/ml]^a^	–	6.22 (4.79–22.88)	7.61 (5.22–41.89)	^***^	6.25 (4.89–59.55)	7.73 (4.83–51.50)	^**^^b^^,^ ^c^	6.67 (4.80–20.51)	7.07 (4.81–52.24)	
IGF-1 [ng/ml]	–	131.20 (48.72)	134.80 (42.82)		133.50 (40.02)	141.79 (36.72)		161.57 (44.16)	168.05 (38.74)	
sqrtVEGF [pg/ml]^a^	–	4.31 (3.02–34.34)	4.34 (2.86–38.69)		4.56 (2.92–70.42)	5.29 (3.29–74.47)		3.52(3.42–3.66)	4.12(3.10–5.26)	

*Physical fitness was assessed with the Senior Fitness Test from Rikli and Jones (2001). The p-values are shown for the post-hoc pairwise comparisons. BDNF, brain-derived neurotropic factor, CT, Cognitive training, CPT, Cognitive training with additional physical activity; CPT+C, Cognitive training with additional physical activity and counseling; IGF-1, insulin-like growth factor 1; ng/ml, nanograms per milliliter; pg/ml, pictograms per milliliter. sqrt, square-root transformation to handle extreme outliers; VEGF, vascular endothelial growth factor*.

a *Medians and Ranges are only displayed for the variables that had to be analyzed with non-parametric methods*.

b *p-values of comparison 1 (CPT vs. CT)*.

c * p-values of comparison 2 (CPT vs. CPT+C)*.

* *p ≤ 0.05;*

** *p ≤ 0.01;*

*** *p ≤ 0.001*.

#### Primary outcomes in neuropsychological performance

For analysis of the differences between groups in primary outcomes we used rANOVAs with the within-subject variable *Time* (pre- vs. post-test) and the between-subject variable *Training*. Significant interaction effects show differences in training gains. *Post-hoc* tests of the within-subject variable *Time* indicated cognitive changes for each group separately. Equivalent non-parametric tests were used where appropriate.

##### Testing hypothesis 1: CPT vs. CT

**Figure 2:**
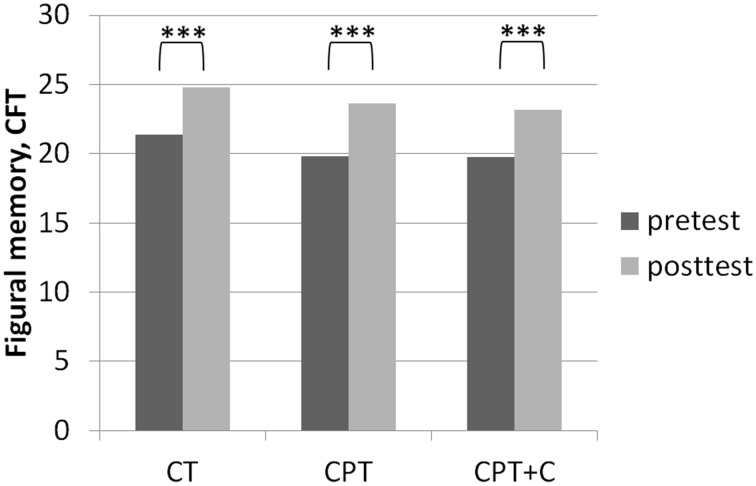
**Results of *post-hoc* analyses of within-subject factor Time for figural memory assessed with the Complex Figure Test (CFT) as described in Strauss et al. (2006) in each training group separately**. ^***^p ≤ 0.001.

**Figure 3:**
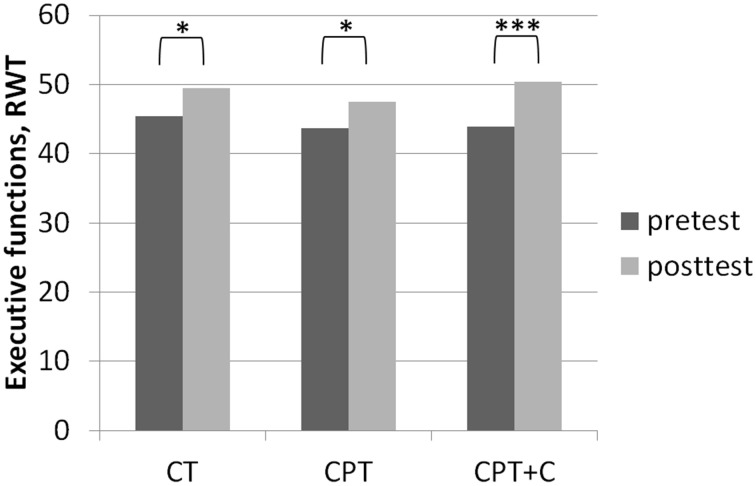
**Results of *post-hoc* analyses of within-subject factor Time for executive functions assessed with the Regensburger Wort Flüssigkeits-Test (RWT, Aschenbrenner et al., 2000) in each training group separately**. ^*^p ≤ 0.05; ^***^p ≤ 0.001.

No significant interaction effects *Time* × *Training* were found when comparing CPT vs. CT. Overall analyses revealed significant within-subject effects of *Time* for figural memory, *F*_(1, 43)_ = 29.11, *MSE* = 9.89, *p* = 0.00, η^2^_p_ = 0.40 (see Figure 2), working memory, *F*_(1, 43)_ = 6.11, *MSE* = 2.35, *p* = 0.02, η^2^_p_ = 0.12, letter verbal fluency, *F*_(1, 43)_ = 9.32, *MSE* = 37.07, *p* = 0.00, η^2^_p_ = 0.18 (see Figure 3), and cognitive state, *F*_(1, 43)_ = 8.27, *MSE* = 1.89, *p* = 0.04, η^2^_p_ = 0.09. *Post-hoc* analyzes of effects from pre- to post-test of each intervention separately revealed different effects for the CPT vs. CT group: While both CPT and CT significantly improved figural memory, CPT: _mean_diff = 3.80, *p* = 0.00, *d* = 0.96 vs. CT: _mean_diff = 3.40, *p* = 0.00, *d* = 0.68, and letter verbal fluency, CPT: _mean_diff = 3.88, *p* = 0.03, *d* = 0.41 vs. CT: _mean_diff = 4.00, *p* = 0.04, *d* = 0.53, only CPT led to gains in working memory, _mean_diff = 1.00, *p* = 0.03, *d* = 0.45. The effects for cognitive state did not reach significance in pairwise comparisons anymore.

##### Testing hypothesis 2: CPT vs. CPT+C

**Figure 4:**
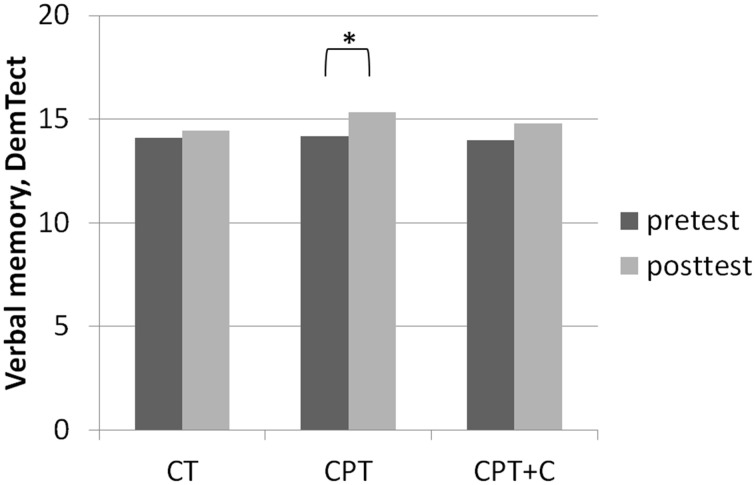
**Results of *post-hoc* analyses of within-subject factor Time for verbal memory assessed with the DemTect (Kalbe et al., 2004) in each training group separately**. ^*^p ≤ 0.05.

A significant interaction effect *Time* × *Training* was found in favor of CPT+C for alternating letter verbal fluency, *F*_(1, 46)_ = 5.31, *MSE* = 489.28, *p* = 0.03, η^2^_p_ = 0.10. Overall analyses revealed significant within-subject effects of *Time* for verbal memory, *F*_(1, 46)_ = 5.51, *MSE* = 4.10, *p* = 0.02, η^2^_p_ = 0.11 (see Figure 4), figural memory, *F*_(1, 46)_ = 34.60, *MSE* = 9.06, *p* = 0.00, η^2^_p_ = 0.43 (see Figure 2), attention, *F*_(1, 46)_ = 6.17, *MSE* = 3.48, *p* = 0.02, η^2^_p_ = 0.12, letter verbal fluency, *F*_(1, 46)_ = 16.96, *MSE* = 37.90, *p* = 0.00, η^2^_p_ = 0.27 (see Figure 3), and planning, *F*_(1, 46)_ = 10.08, *MSE* = 3.67, *p* = 0.00, η^2^_p_ = 0.18. A significant between-group difference was found for planning, *F*_(1, 46)_ = 8.77, *MSE* = 3.62, *p* = 0.01, η^2^_p_ = 0.16. *Post-hoc* analyzes of effects from pre- to post-test of each intervention separately revealed that both CPT and CPT+C showed improvement in figural memory, CPT: _mean_diff = 3.80, *p* = 0.00, *d* = 0.96 vs. CPT+C: _mean_diff = 3.44, *p* = 0.00, *d* = 0.75, and letter verbal fluency, CPT: _mean_diff = 3.88, *p* = 0.03, *d* = 0.41 vs. CPT+C: _mean_diff = 6.48, *p* = 0.00, *d* = 0.82. Only CPT further improved verbal memory, _mean_diff (post—pre) = 1.16, *p* = 0.05, *d* = 0.53, and attention, _mean_diff = 1.24, *p* = 0.02, *d* = 0.46, and solely the CPT+C group showed pre-post-improvements in planning, _mean_diff = 1.57, *p* = 0.01, *d* = 0.61. At posttest the between-group difference of CPT+C vs. CPT was significant in planning with higher scores for CPT+C, _mean_diff = 2.38, *p* = 0.00, *d* = 1.09.

#### Secondary outcomes

For analysis of the differences between groups in secondary outcomes we used rANOVAs with the within-subject variable *Time* (pre- vs. post-test) and the between-subject variable *Training*. Significant interaction effects show differences in training gains. *Post-hoc* tests of the within-subject variable *Time* indicated cognitive changes for each group separately. Equivalent non-parametric tests were used where appropriate.

##### Physical fitness

###### Testing hypothesis 1: CPT vs. CT

No significant interaction effects *Time* × *Training* were found when testing hypothesis 1. Significant within-subject effects of *Time* in overall analyses were found for each of the Seniors Fitness subtests (all *p* ≤ 0.04). rANOVAs revealed a significant between-group effect for overall fitness, *F*_(1, 43)_ = 5.97, *MSE* = 459.37, *p* = 0.02, η^2^_p_ = 0.12. Significant between-group effects at posttest were found for upper, *H*_(1)_ = 15.28, *p* = 0.00, ϕ = 2.28, and lower body strength, *H*_(1)_ = 8.84, *p* = 0.00, ϕ = 1.32, in non-parametric analyses. In detail, CPT improved overall fitness, _mean_diff = 13.32, *p* = 0.00, *d* = 1.22, upper body strength, *z* = −3.24, *p* = 0.00, ϕ = −0.65, lower body strength, *z* = −3.35, *p* = 0.00, ϕ = −0.67, flexibility, _mean_diff = 17.16, *p* = 0.00, *d* = 0.67, coordination, _mean_diff = 9.52, *p* = 0.00, *d* = 1.00, and endurance, _mean_diff = 10.14, *p* = 0.03, *d* = 0.43. CT only improved overall fitness, _mean_diff = 6.74, *p* = 0.01, *d* = 0.55. Remarkably, CT showed significant less overall fitness, upper, and lower body strength when compared to CPT at posttest (all *p* = 0.01).

###### Testing hypothesis 2: CPT vs. CPT+C

No significant interaction effects *Time* × *Training* were found when testing hypothesis 2. Significant within-subject effects of *Time* in the overall analyses were found for each of the Seniors Fitness subtests (all *p* ≤ 0.04).

Again and comparable to the results from hypothesis 1, CPT improved all fitness parameters (all *p* = 0.00), but improvement in endurance only came close to significance in this comparison, _mean_diff = 10.14, *p* = 0.06, *d* = 0.43. The CPT+C group improved overall fitness, _mean_diff = 11.29, *p* = 0.00, *d* = 0.75, upper body strength, *z* = −3.40, *p* = 0.00, ϕ = −0.71, lower body strength, *z* = −3.00, *p* = 0.00, ϕ = −0.63, and coordination, _mean_diff = 9.32, *p* = 0.00, *d* = 0.58.

##### Peripheral growth factors

Due to extreme outliers in peripheral blood BDNF and VEGF levels at pre- and post-test, the values of these parameters were transformed with square-root transformation and non-parametric analyses were used for estimation of effects. Furthermore, the detection limit of the VEGF-A ELISA was set at 7.9 pg/ml, which means that all values below this cut-off were considered as not differing from zero. Due to this, the VEGF data of only *n* = 22 participants could be analyzed (CPT: *n* = 15, CPT+C: *n* = 3, CT: *n* = 4).

###### Testing hypothesis 1: CPT vs. CT

No interaction effects *Time* × *Training* were found. Overall analyses revealed significant increases of BDNF from pre- to post-test, χ^2^ = 11.76 (1), *p* = 0.00, ω = 0.26. *Post-hoc* analyses indicated that both interventions increased peripheral BDNF, CPT: *z* = −2.54, *p* = 0.01, ϕ = −0.51, CT: *z* = −3.02, *p* = 0.00, ϕ = −0.68.

###### Testing hypothesis 2: CPT vs. CPT+C

No interaction effects *Time* × *Training* were found. Overall analyses revealed significant increases of BDNF, χ^2^ = 4.08 (1), *p* = 0.04, ω = 0.58, and IGF-1, *F*_(1, 46)_ = 3.99, *MSE* = 327.54, *p* = 0.05, η^2^_p_ = 0.08, from pre- to post-test. *Post-hoc* analyses indicated that solely CPT increased blood levels of BDNF, *z* = −2.54, *p* = 0.01, ϕ = −0.51, and blood levels of IGF-1 approached significance only in CPT, _mean_diff = 8.29, *p* = 0.11, *d* = 0.36.

### Predictors of cognitive improvement within CPT

**Figure 5:**
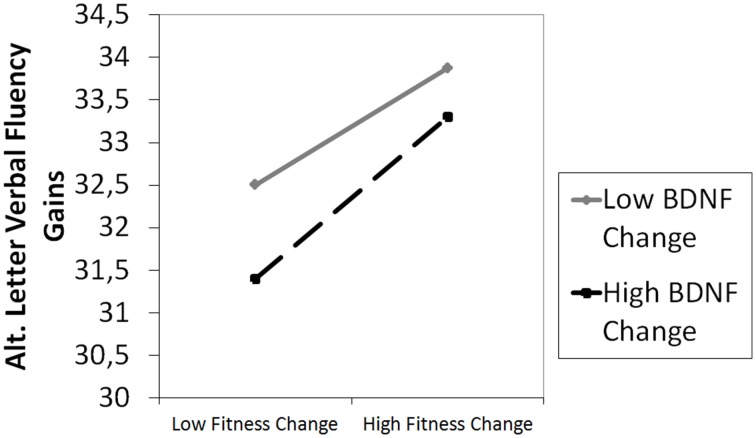
**Moderation of the effect of change in overall physical fitness on gains in alternating (alt.) letter verbal fluency by change in BDNF (β = 0.37, *p* = 0.04)**. High changes in overall fitness were predictive for high cognitive gains (β = 0.31, *p* = 0.07). This positive association was moderated by the BDNF change and strengthened with higher change scores in BDNF. However, participants with lower BDNF change scores showed higher gains in alternating letter verbal fluency than those with higher change scores.

To analyze predictors of cognitive improvement within CPT we used backwards multiple regressions to ensure achievement of best model fit while taking into account each relevant predictor. Assuming a maximum of five predictors per model (based on *n* = 25 participants in the CPT group), this study had 94% power to detect predictors of a model with at least R^2^ = 0.50 (α = 0.05) in the CPT group. The main results of the predictor analyses are: (i) low baseline performance was a predictor for gains in verbal memory (β = −0.57), figural memory (β = −0.66), attention (β = −0.58), working memory (β = −0.40), and alternating letter verbal fluency (β = −0.42), (ii) low blood levels of BDNF were a predictor for improvement in verbal memory (β = −0.34) and letter verbal fluency (β = −0.29), (iii) a negative apoE4 carrier status was predictive for improvement in alternating letter verbal fluency (β = −0.33), and (iv) improvement in physical fitness was a predictor for gains in attention (β = −0.29) and alternating letter verbal fluency (β = −0.38). Given the results of the prediction in performance of alternating letter verbal fluency, we considered the interaction of *change in physical fitness* × *change in BDNF* on alternating letter verbal fluency and modeled changes in alternating letter verbal fluency a second time based on the predictors apoE4 carrier, baseline performance in (i) alternating letter verbal fluency, (ii) physical fitness, change in (i) BDNF, (ii) physical fitness, and the interaction *change in physical fitness* × *change in BDNF*. Results of this second analysis showed that the interaction of *change in physical fitness* × *change in BDNF* predicted an improvement in alternating letter verbal fluency (β = −0.37), indicating a moderation of the effects of change in physical fitness on cognition by change in BDNF. This moderation effect is plotted in Figure 5. Statistical details are shown in Table 1 of the Supplementary Material.

## Discussion

The main findings of our study are that (i) although both CPT and CT lead to significant cognitive improvements and an increase of peripheral BDNF from pre- to post-test, no significant interaction effect which could support hypothesis 1—that CPT is superior to CT—could be found. However, the facts that CPT and CT showed diffuse patterns of improved domains with CPT in sum affecting more cognitive domains including working memory, and that only CPT improved all physical fitness domains point to some advantages of this training; beyond that, the effects of both interventions were greater than common retest effects which further underlines the clinical relevance of our results; (ii) a significant interaction effect in favor of CPT+C compared to CPT for alternating letter verbal fluency as well as a significant enhancement of planning abilities (both executive functions) only after CPT+C was found; although at first sight this result seems to support hypothesis 2—that CPT with additional counseling is more efficient in enhancing physical activity and, in the second place, in improving cognition—the effects are not explainable by the intended stronger improvements of physical fitness, as CPT was even superior in this aspect; furthermore, only CPT additionally enhanced verbal memory, attention, and also peripheral BDNF from pre- to post-test; thus, CPT in total seems at least as efficient as CPT with additional counseling; (iii) improvement of cognitive functions after CPT can significantly be predicted by neuropsychological baseline performance, baseline level of BDNF, APOE polymorphism, improvement in physical fitness, and the moderation of the effects of improvement in physical fitness by change in BDNF.

### Discussion of hypothesis 1: CPT vs. CT

The fact that no significant interaction effects in favor of CPT in comparison to pure CT could be observed contradicts our hypothesis 1 that CPT is superior and is in line with other RCTs failing to support the superiority of CPT (Legault et al., 2011; Barnes et al., 2013; Shatil, 2013; Linde and Alfermann, 2014). Remarkably, besides the question whether one or the other training is superior, it should be emphasized that both CPT and CT can be regarded as efficient strategies to stabilize or even enhance cognitive functions in healthy older adults (Kelly et al., 2014; Law et al., 2014). In our study, both trainings yielded significant within-subject effects in memory (figural memory, see Figure 2) and executive functions (letter verbal fluency, see Figure 3)—both functions that are prone to aging-associated cognitive decline (Kemps and Newson, 2006; Singh-Manoux et al., 2012). The gains in figural memory after CT are in line with previous findings (Peretz et al., 2011). Also congruent with our results after CPT, Erickson et al. (2009) reported a triple interaction between higher fitness levels, larger hippocampi, and better spatial memory performance. Furthermore, the improvement in verbal fluency (i.e., executive functions) is in line with other findings that executive functions are improved after CT (e.g., Nouchi et al., 2012) and physical training (see Colcombe and Kramer, 2003; Erickson et al., 2012b).

The fact that not only CPT, but also CT improved overall physical fitness, was unexpected. To interpret this result, one should keep in mind that the participants were not blinded for the three intervention groups, and some participants in CT reported disappointment about the lack of physical activity in their intervention. Albeit speculative, it seems possible that higher physical fitness in this group was the result of an intervention-independent attempt to improve physical activity next to CT—even though the participants were instructed to remain physically active at their usual level. In line with the findings that physical fitness was enhanced in both groups, also BDNF, a neurobiological marker of cerebral plasticity in healthy older adults, increased in both groups. While this was expected for the CPT group, as increases of peripheral BDNF after physical activity are well established (e.g., Coelho et al., 2013), to our knowledge this is the first study demonstrating that also a pure CT lead to increases in BDNF. Whether this latter result is associated to the improvement of physical fitness in this group or a direct consequence of the CT will have to be subject to further investigations.

### Discussion of hypothesis 2: CPT vs. CPT+C

A significant interaction effect in alternating letter verbal fluency favoring CPT+C as well as a pre- vs. post-test enhancement of planning only in the CPT+C group speak in favor of our hypothesis that additional counseling leads to an additional cognitive benefit of the training. However, some other results show that the pattern must be regarded more differentially. First, CPT improved more cognitive domains in the pre- vs. post-tests within comparisons than CPT+C [with only CPT yielding enhancement in the important domains of verbal memory (see Figure 4) and attention]. Second, only the CPT group improved in all physical fitness domains, while the CPT+C showed improvements of only strength, coordination, and overall physical fitness. This finding is specifically astonishing, as counseling was meant to optimize physical activity (and indirectly show an additional benefit on cognition). Finally, BDNF only increased in the CPT group, and there was even a trend for improvement in IGF-I—a finding that is congruent with the fact that CPT enhanced physical fitness stronger than CPT+C, as an increase of BDNF is well established after physical activity (e.g., Coelho et al., 2013), and IGF-1 is produced in the muscles of the periphery (Liu-Ambrose et al., 2012).

Although speculative, it seems as if the two training groups were involved in two different ways: The group without counseling was involved on the behavioral level (being physically active), while the group with counseling was involved on a more cognitive level (planning physical activity efficiently). This would explain the improvement in the cognitive domains of planning and alternating letter verbal fluency—both sub-functions of the executive domain—in the CPT+C group. Especially verbal fluency has been reported to be highly affected by the use of cognitive strategies (Hughes and Bryan, 2002), and the alternating letter verbal fluency task requests specifically high executive demands (Aschenbrenner et al., 2000). Although this can only be reported as anecdotal evidence, this interpretation seems to fit to complaints of several participants of the CPT+C group about their higher expenditure of time and strains caused by the additional individual counseling sessions as well as the work with the individual fitness plan. Compared to CPT without counseling, these intervention components came on top of the group training. Furthermore, the counseling focused on motivation, and cognitive strategies to improve motivation for physical activity were used in the appointments. The task of the participants was to transfer the cognitive strategies in their everyday lives and—importantly—in their physical behavior. However, the results in physical fitness variables and with regard to the BDNF level indicate that the transfer was inefficient. Thus, it is possible that the participants' complaints about higher strains through the training might have resulted in less training motivation and, consequently, less training. Interestingly, the drop-out rate in this group was the highest of all three interventions (CT 8.70%, CPT 10.71%, CPT+C 23.33%). Thus, it can be summarized that a concrete training plan which participants can follow without additional individual effort might be even more efficient than training with additional counseling. As “personal training” is a method that is increasingly used by individuals further studies are needed whether this method may be more efficient in healthy adults who have a higher motivation for individual optimization of their training.

### Discussion of predictor analysis within CPT

In line with evidence from interventions aiming at an improvement of cognitive functions (Whitlock et al., 2012), low baseline scores in the specific cognitive domains were predictive for improvements in these domains. However, contradictory results exist (e.g., Fairchild et al., 2012). Interestingly, lower blood levels of BDNF were a predictor for improvement in verbal memory and letter verbal fluency, suggesting that at least for these two verbal domains the initial blood BDNF level was important for training induced plasticity. This result is at odds with observations reported by Voss et al. (2013a) who found that higher levels of BDNF at baseline were associated with greater functional connectivity change in a group trained with non-aerobic exercises. However, Voss et al. (2013a) did not assess the relationship between growth factors and cognitive gains. Furthermore, the intervention used by Voss et al. (2013a) and the one used in the current study are not comparable as we used a combined CPT targeting both cognitive and physical activity. Future studies need to further investigate the role of initial levels of growth factors for training induced cognitive improvement following different intervention types.

The APOE polymorphism was predictive solely in the domain of alternating letter verbal fluency, possibly because this task was the most demanding of our test battery. We investigated healthy older adults with age-adequate overall cognitive performance (>12 points in the DemTect, Kalbe et al., 2004); accordingly, the strongest influence of this polymorphism on cognitive performance is to be expected in challenging tasks with high difficulty. Most consistently, effects of the APOE polymorphism on cognition and etiopathology have been described for patients suffering from Alzheimer's disease (Liu et al., 2013), but it's effects on cognitive functions in healthy older adults has also been reported (Wisdom et al., 2011). Remarkably, to our knowledge, there is only one study reporting effects of the APOE polymorphism on CT gains after a cognitive intervention in patients with Mild Cognitive Impairment (MCI) or dementia (Binetti et al., 2013), but so far no evidence has been reported that the APOE polymorphism affects CT gains in healthy older adults. Therefore, future research has to focus on this topic and to investigate which role this specific genetic polymorphism plays when training healthy older adults with different single and combined cognitive interventions.

In good accordance with our interpretation that the stronger improvements in physical fitness in the CPT group can be associated with stronger cognitive improvements, we found improvement in physical fitness to be predictive for training effects in attention and alternating letter verbal fluency. Furthermore, we analyzed whether the interaction of *change in growth factors* × *change in physical fitness* might predict cognitive improvements. We found the interaction of *change in physical fitness* × *change in BDNF* to be a significant predictor of gains in alternating letter verbal fluency indicating a moderation effect. After a single assessment of recognition memory accuracy, serum resting BDNF, and aerobic fitness operationalized by VO_2_ max tests, Whiteman et al. (2014) reported an association between BDNF level and cognitive performance in a sample of healthy young adults that was moderated by aerobic fitness, in the way that BDNF had a negatively predictive value for recognition accuracy at low fitness and a positive predictive value at high fitness. Note, however, that when an interaction term is a significant predictor in a regression analysis, the direction of the association can be interpreted in both directions. Therefore, our data suggest an alternative interpretation of this interaction effect, because (i) in our second regression model BDNF change was not a significant predictor anymore, but change in overall physical fitness and the interaction term were significant predictors and (ii) based on the literature (e.g., Coelho et al., 2013) we assumed that the BDNF change would be a consequence of improved physical fitness and therefore any BDNF change seems more likely to be the moderator than the independent variable in the found interaction term. Hence, we interpret the significant interaction as the moderation of the effect of change in overall physical fitness on gains in alternating letter verbal fluency by change in BDNF, indicating that high changes in physical fitness were predictive for high cognitive gains (a similar positive association was found for attention) and a strengthening of this association with higher BDNF changes (see Figure 5). However, Figure 5 also visualizes that the highest cognitive gains were found for participants who showed low BDNF change scores. At a first glimpse, this unexpected negative association between change in BDNF and cognitive gains seems not in line with our finding of increased BDNF at a group level as well as evidence supporting the beneficial effects of physical activity on peripheral BDNF (e.g., Lista and Sorrentino, 2010; Coelho et al., 2013). However, an inverse relationship between cardio-respiratory fitness and peripheral BDNF has been reported (Currie et al., 2009) and BDNF levels were found to be lower in highly trained vs. moderately trained (Chan et al., 2008) or sedentary subjects (Nofuji et al., 2012). Referring to these results, Huang et al. (2014) argued that peripheral BDNF clearance in form of storage, transport to the brain, or use in the periphery might be more effective in higher trained subjects. Therefore, we interpret the finding that participants with lower BDNF change showed higher gains in alternating letter verbal fluency in line with the assumption of Huang et al. (2014) and suppose that a subgroup of all participants already had a better BDNF clearance and therefore in those participants possibly more BDNF was available for cognitive and neuronal plasticity processes explaining their overall higher gains in alternating letter verbal fluency. Even though our interpretation is speculative, our findings are striking as they support the notion that individual differences in physiological profiles impact upon plasticity (cf. Voss et al., 2013a). Thus, our findings underline the importance of the association between changes in physical fitness and changes in BDNF and cognitive gains which should be investigated in more detail in future studies. The identification of predictors of cognitive interventions has gained increasing interest as information on who will benefit from such interventions might help to identify appropriate target groups for future research. Furthermore, such information might be useful when optimizing interventions to be most effective in specific target groups and especially might help to develop alternatives for those who might possibly need other intervention strategies. Future studies will have to further investigate the meaning of predictors for training success of CPT.

### Limitations

Some limitations have to be considered when interpreting the current results. First, participants were highly educated, active in several stimulating leisure activities, and no homozygote E4/E4-carrier was identified. To some degree, our narrow inclusion and exclusion criteria might have limited the variability in our sample, e.g., it can be expected that homozygote E4/E4-carrier who have the highest risk to develop cognitive dysfunction or dementia (Liu et al., 2013) may have been excluded as a result of our screening with the DemTect (Kalbe et al., 2004). Furthermore, the self-selection bias of older adults participating in a training study is caused to some part by the general problem that volunteers might differ in motivation, outcome expectation, socio-demographic variables, or healthy lifestyles (Oswald et al., 2006; Unverzagt et al., 2009; Schubert et al., 2014; Rahe et al., 2015b).

Second, at pretest, blinding of neuropsychological and physical fitness test leaders was perfect as no one knew the training group affiliation of participants. However, at posttests blinding realization was much harder as the trainer of the intervention groups had to perform some assessments due to staffing shortage. Furthermore, participants reported about their interventions even though they were instructed not to give clues to test leaders. This lack of blinding at posttest could also account for the observation that CT improved physical fitness unexpectedly. In future, blinding should be optimized and ideally test leaders should even be blinded for time of assessment (pre- vs. post-test). Fortunately, the analyses of growth factors can be evaluated as free of any test leader expectation as blood analyses were performed in laboratories with study-independent staff who did not even know the time of assessment.

Third, very few interaction effects Time × Group were found. Nevertheless, we are convinced that our results in comparing effects of the different types of intervention in *post-hoc* analysis are “real” and meaningful. Importantly, as the focus of this study lay on the hypothesis that the combination of CT with other lifestyle factors is superior to pure CT, we did not include a passive control group for comparisons. However, one could argue that pre- to post-test effects could be derived to retest effects or falsification provoked by the participants' knowledge of the neuropsychological tests at posttest—especially for those domains in which pre- to post-test effects were found for all three interventions (figural memory and letter verbal fluency, see Figures 2, 3). However, consideration of retest-effects reported in test manuals in comparison to change scores in our study show that this is unlikely: For example for the *delayed recall* of the CFT (figural memory, Strauss et al., 2006) mean gains of 2.30 points with an *r* = 0.79 haven been reported, while the gains in figural memory of the three groups in our study ranged between 3.40 points (CT), 3.43 points (CPT+C), and 3.80 points (CPT) and thus were higher (see Figure 2). Finally, the fact that the pattern of improvement in other domains induced by the three interventions is different between the three intervention groups further argues against retest effects (e.g., verbal memory, see Figure 4).

Fourth, leisure physical activity (namely activity outside the experiment) was not controlled for in the analyses. However, participants were instructed to remain physically active at their usual level. In future studies, this short-coming should be addressed by assessing leisure physical activity, e.g., with self-report questionnaire or a physical activity diary during the training period.

Fifth, blood sampling and preanalytics for blood analyses were performed in three different laboratories and afterwards samples were sent to three laboratories where levels of IGF-1 (Central Laboratory of the University Hospital Cologne, Germany), levels of BDNF and VEGF (MVZ Laboratory Dr. Eberhard and Partner in Dortmund, Germany), or APOE and BDNF polymorphisms (Institute of Human Genetics, University Hospital Cologne, Germany) were analyzed. Blood sampling and preanalytics were standardized in all study centers, but a bias due to different laboratory settings cannot be excluded entirely. For example, blood sampling was performed at different day times to limit disturbance of the daily routines in the laboratories. Nevertheless, within the three study centers blood sampling at pre- and post-tests were performed at the same day time, so that the effect of variations in neurotrophic blood levels due to circadian rhythmicity was presumably hold constant within subjects. Furthermore, we instructed participants not to be physically active the last 24 h before blood sampling to control for the acute effect of physical activity on the growth factors so that variance of errors can be at least partly ruled out. Furthermore, we analyzed BDNF and VEGF blood levels after square-root transformation of the values to minimize the effects of outliers. Nevertheless, in future studies time of blood withdrawal should be standardized to rule out effects of circadian rhythmicity on neurotrophic growth factors (Begliuomini et al., 2008; Piccinni et al., 2008; Choi et al., 2011), and other determinants of neurotropic factors, such as sex, should be included in the analyses as both Piccinni et al. (2008) as well as Choi et al. (2011) reported sex-related rhythmic variations in plasma BDNF with higher BDNF levels in the morning only in men, but no diurnal variations in women. Furthermore, Pillai et al. (2012) and Choi et al. (2011) found associations between body weight and BDNF levels. For the present study sex-specific analyses would be largely underpowered so that this must be addressed in future studies. Finally other factors such as cortisol seem to co-regulate BDNF circadian trends (Begliuomini et al., 2008) and should be controlled for in future studies as well.

As a final limitation, the Chemilumineszenz-Immunoassay (Siemens, Germany) for the IGF-1 analyses was no longer deliverable in January 2013 and therefore the Sandwich-Chemilumineszenz-Immunoassay (DiaSorin, Italy) was used for the further analyses. Fortunately, this change fell between two waves of data collection and therefore the pre- and post-tests of all participants were analyzed with the same immunoassay. Furthermore, comparability of IGF-1 levels between the two immunoassays was confirmed by the laboratory. Future studies should strive to standardize preanalytics and blood analyses completely.

## Conclusion

Even though all three interventions used in this study led to cognitive improvements, our results do not support the notion that CPT or CPT+C with counseling have additional benefit in improving cognitive functions of healthy older adults when compared to pure CT. Therefore, both single and combined interventions can be recommended. However, as both CT and physical activity positively impact cognition (Erickson et al., 2012b; Kelly et al., 2014), but only physical activity has additional benefit on cardiovascular risk factors (and also yielded more effects on physical fitness in our study)—which in turn have been reported to have influence on cognition as well (Fillit et al., 2008) - there are arguments that for general health improvement CPT seems to be more advantageous and therefore seems especially effective.

Precondition for positive effects of both CT or CPT interventions are cognitive and neuronal plasticity mechanisms which have often been described within the concepts of cognitive and/or brain reserve (Stern, 2009; Pieramico et al., 2014). A large body of studies addressed the meaning of cognitive and/or brain reserve in aging and neuropsychiatric diseases such as depression (Freret et al., 2015). It has already been argued that physical and cognitive activities seem important protective factors in aging and their role for brain and cognitive reserve has been discussed as well (Chapman et al., 2013; Huckans et al., 2013; Schneider and Yvon, 2013; Law et al., 2014; Pieramico et al., 2014). In line with these studies our results show that both CT and CPT can be sufficient strategies to enhance cognitive and brain reserve.

Furthermore, as our predictor analyses revealed that improvements in physical fitness were predictive for cognitive improvements in attention and executive functions, further research to test the hypothesis that CPT might show superior cognitive effects compared to CT still seems justified. Maybe, the superiority of CPT compared to CT might not become apparent before follow-up assessments as it has been reported in another recent study (Rahe et al., 2015b). In line with this assumption, a recent study found the positive effects of cognitive activities to be apparent for a shorter follow-up period (5 years) compared to the positive effects of physical activities (10 years; Jedrziewski et al., 2014). Thus, the impact of combined CPT interventions on prevention of cognitive decline and the duration of training effects need to be addressed in future studies. Moreover, the results of future RCT with long-term data assessment will have to shed further light on the effects and mechanisms of non-pharmacological interventions to stabilize cognition and delay cognitive decline in healthy elderly individuals.

### Conflict of interest statement

Dr. Julia Rahe, Prof. Elke Kalbe, and Prof. Josef Kessler are authors of the forthcoming NEUROvitalis Plus program. Prof. Elke Kalbe and Prof. Josef Kessler are authors of the NEUROvitalis program. This study was part of the PhD project and thesis of Dr. Julia Rahe. The authors declare that the research was conducted in the absence of any commercial or financial relationships that could be construed as a potential conflict of interest.

## Abbreviations

CPT: cognitive-physical training
CPT+C: cognitive-physical training with counseling
CT: cognitive training.
