# Supplementary material for: Cognitive training with and without additional physical activity in healthy older adults: cognitive effects, neurobiological mechanisms, and prediction of training success
Source: Front Aging Neurosci. 2015 Oct 13;7:187. doi: 10.3389/fnagi.2015.00187 (PMC4602086; doi:10.3389/fnagi.2015.00187)
Supplement: Supplementary file 1 [file Presentation1.PDF]

Supplementary Material

Description of the three interventions

Figure 1 provides an overview of the structure and components of these interventions.

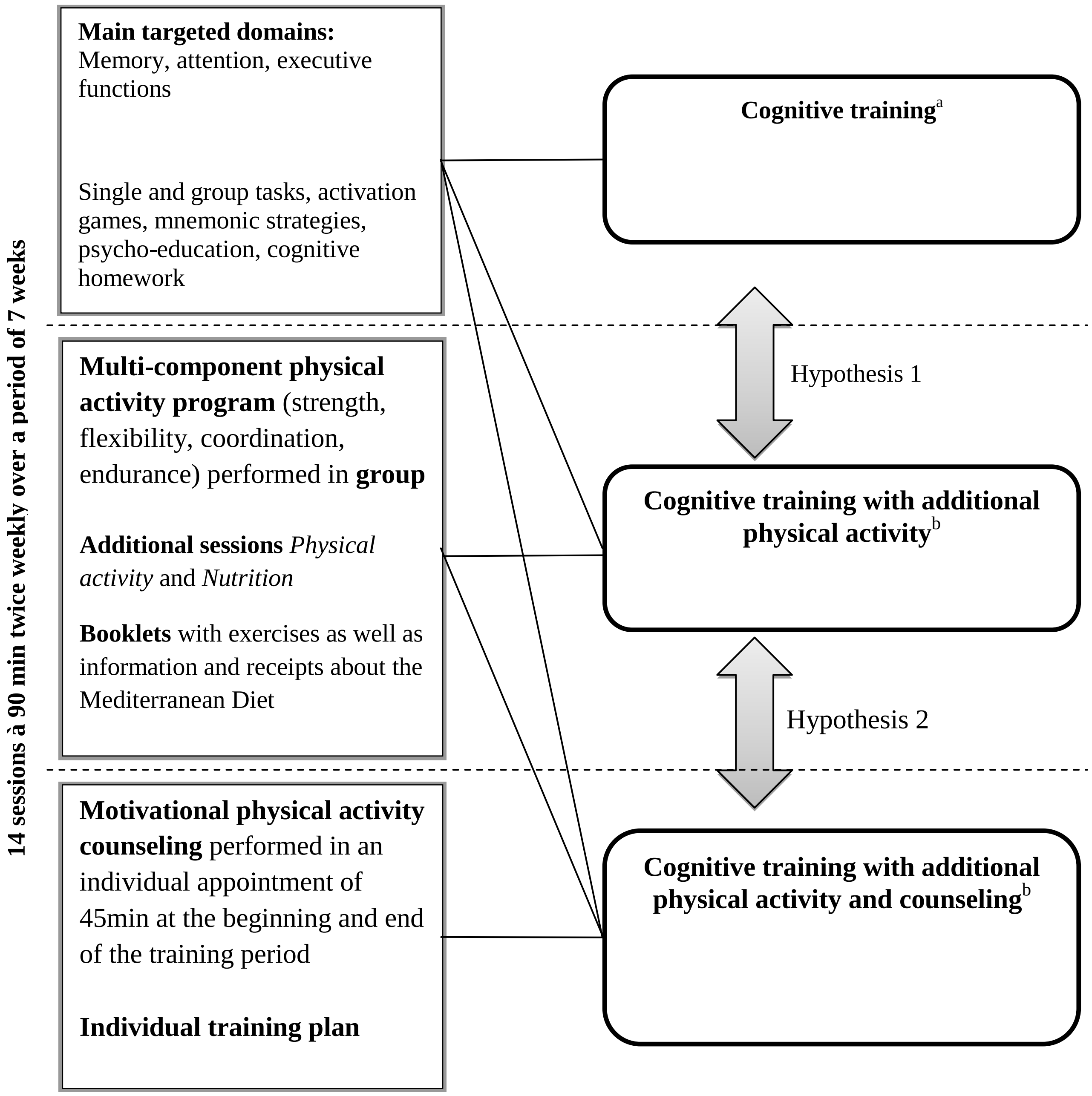

Figure 1. Comparison of the three interventions and the training components.

[a] Two repetition session were added to the pure cognitive training to ensure an equal amount of training sessions between the three intervention groups.

[b] The physical activity program was performed during the first 20 min of each session.

Pure Cognitive Training

The neuropsychological program NEURO*vitalis* (Baller et al., 2009) was used for CT. NEURO*vitalis* specifically targets the aging-sensitive domains memory, attention, and executive functions. Furthermore, the training covers mnemonic strategies for compensation of mnestic deficits. In each session participants performed activation games, single, and group tasks to exercise cognitive functions. Furthermore, each session included a psycho-educational part with topics such as *Relevance of attentional processes*, *How does memory work?*, or *Planning and problem solving*. Additionally, participants were asked to perform cognitive homework for ten minutes each day. Due to the two additional sessions in the CPT groups, NEURO*vitalis* was supplemented with two repetition sessions, integrated as the 6th and 12th sessions. NEURO*vitalis* has been shown to yield short-term and long-term cognitive gains in non-demented patients with Parkinson´s disease (Petrelli et al., 2014; Petrelli et al., 2015), MCI patients (Rahe et al., 2015a) as well as in healthy older adults (Rahe et al., 2015b).

Cognitive Training with Additional Physical Activity

The standardized NEURO*vitalis* Plus training was used as CPT with a special focus on cognitive training with additional physical activity. A former version of the CPT has been investigated in a recent study (Rahe et al., 2015b). The CPT of this RCT comprised the CT (see 2.2.1) but was supplemented by the sessions *Physical Activity* (2nd session) and *Nutrition* (3rd session) as well as a multi-component physical activity program (strength, flexibility, coordination, endurance) over the course of the training sessions. The session *Physical activity* covered physical activity recommendations for older adults (adapted from Nelson et al., 2007), strategies to increase physical activity in everyday life such as taking the stairs instead of the elevator, and information on positive effects of physical activity on both body and brain health. For practicing at home, the participants received a booklet which illustrated the exercises performed during the training sessions. CPT also targeted nutritional aspects: In the session *Nutrition* the Mediterranean diet (cf. Scarmeas et al., 2006; Scarmeas et al., 2009) was highlighted and participants received a booklet with information and recipes. In each CPT session, the physical and cognitive parts were trained in a consecutive manner: The physical activity program was performed during the first 20 minutes of each session starting with moderate walking or seated aerobic warm-up.

Cognitive Training with Motivational Physical Activity Counseling

The CPT with physical activity counseling (CPT+C) followed the same structure as the CPT, but in addition participants were invited to physical activity counseling. Counseling was performed in the first and the last training week according to the approaches of Biddle and Mutrie (2008) and Marcus and Forsyth (2003). Based on the results of the fitness test at pretest, a stage-dependent training schedule with individual exercises, activities, and motivation strategies was generated for each participant. The schedule targeted strength, flexibility, coordination, and endurance. At an individual appointment in the first week of the intervention, the trainer discussed the training schedule with each participant and together they developed goals for both half-term and the end of the training as well as strategies to attain those goals. At the end of the program, the trainer and the participant checked which goals were attained and which exercises, activities, or strategies should be continued by the

participant after the training. The trainer used motivational interviewing techniques (Miller and Rollnick, 2004).

Predictor analyses of CPT's cognitive training success

Table 1. *Backwards Multiple Regression Predicting Cognitive Improvement of the Cognitive Training with Additional Physical Activity*

	Improvement in Verbal Memory	
Predictor	ΔR^2	β
Step1	.52*	
apoE4 Carrier		-.09
Baseline VM		-.46*
Baseline sqrtBDNF		-.25
Baseline PF		-.17
Improvement IGF-1		.26
Step 2	-.01	
Baseline VM		-.49**
Baseline sqrtBDNF		-.27
Baseline PF		-.19
Improvement IGF-1		.24
Step 3	-.03	
Baseline VM		-.55***
Baseline sqrtBDNF		-.31$^+$
Improvement IGF-1		.22
Step 4	-.05	
Baseline VM		-.57***
Baseline sqrtBDNF		-.34*
Total R^2	.39***	
n	25	

	Improvement in Figural Memory	
Predictor	ΔR^2	β
Step1	.48***	
Baseline FM		-.61***
Improvement sqrtBDNF		-.20
Step2	-.04	
Baseline FM		-0.66***
Total R^2	.42***	
n	25	

	Improvement in Attention	
Predictor	ΔR^2	B
Step 1	.52**	
Sex		-.01
apoE4 Carrier		-.08
Baseline A		-.55*
Baseline IGF-1		.01
Improvement PF		.27
Step 2	.00	
apoE4 Carrier		-.08
Baseline A		-.55**
Baseline IGF-1		.01
Improvement PF		.27
Step 3	.00	
apoE4 Carrier		-.08
Baseline A		-.55***
Improvement PF		.27$^+$
Step 4	-.01	
Baseline A		-.58***
Improvement PF		.29$^+$
Total R^2	.47***	
n	25	

Continued

	Improvement in Working Memory	
Predictor	ΔR^2	β
Step 1	.33$^+$	
apoE4 Carrier		.15
BDNF Polymorphism		.21
Baseline WM		-.40*
Baseline PF		.20
Step 2	-.02	
BDNF Polymorphism		.27
Baseline WM		-.40*
Baseline PF		.26
Step 3	-.07	
BDNF Polymorphism		.30
Baseline WM		-.40*
Step 4	-.09	
Baseline WM		-.40*
Total R^2	.12*	
n	25	

	Improvement in Letter Verbal Fluency	
Predictor	ΔR^2	β
Step 1	.56***	
Age		.22
Baseline LVF		-.54***
Baseline sqrtBDNF		-.32*
Improvement IGF-1		.16
Step 2	-.02	
Age		.19
Baseline LVF		-.59***
Baseline sqrtBDNF		-.33*
Step 3	-.03	
Baseline LVF		-.65***
Baseline sqrtBDNF		-.29$^+$
Total R^2	.46***	
n	25	

	Improvement in Alternating Letter Verbal Fluency	
Predictor	ΔR^2	β
Step 1	.48*	
apoE4 Carrier		-.28
Baseline alt. LVF		-.27
Baseline PF		-.05
Improvement PF		.24
Improvement sqrtBDNF		-.30
Step 2	.00	
apoE4 Carrier		-.30$^+$
Baseline alt. LVF		-.26
Improvement PF		.28
Improvement sqrtBDNF		-.30$^+$
Step 3	-.05	
apoE4 Carrier		-.36*
Improvement PF		.30$^+$
Improvement sqrtBDNF		-.38*
Total R^2	.34**	
n	25	

Continued

Predictor	Improvement in Alternating Letter Verbal Fluency with Moderator Effect	
	ΔR^2	β
Step 1	.54*	
apoE4 Carrier		$-.34^{+}$
Baseline alt. LVF		$-.34^{+}$
Baseline PF		.04
Improvement PF		.33
Improvement sqrtBDNF		-.16
Improvement PF x Improvement sqrtBDNF		.30
Step 2	.00	
apoE4 Carrier		$-.33^{+}$
Baseline alt. LVF		$-.35^{+}$
Improvement PF		$.31^{+}$
Improvement sqrtBDNF		-.16
Improvement PF x Improvement sqrtBDNF		.29
Step 3	-.02	
apoE4 Carrier		$-.33^{+}$
Baseline alt. LVF		-.42*
Improvement PF		$.31^{+}$
Improvement PF x Improvement sqrtBDNF		.37*
Total R^2	.43**	
n	25	

Note. A = Attention. alt. = alternating. apoE = Apolipoprotein E. FM = Figural Memory. IGF-1 = insulin-like growth factor 1. LVF = lexical verbal fluency. P = planning. PF = physical fitness. sqrtBDNF = square-root transformed brain-derived neurotropic factor. WM = working memory.
$^{+}$ $p \leq .10$. *$p \leq .05$. **$p \leq .01$ *** $p \leq .001$.

References

Baller, G., Kalbe, E., Kaesberg, S., and Kessler, J. (2009). *NEUROvitalis. Ein neuropsychologisches Gruppenprogramm zur Förderung der geistigen Leistungsfähigkeit.* Köln: ProLog.

Biddle, S.J.H., and Mutrie, N. (2008). *Psychology of Physical Activity.* New York: Routledge.

Marcus, B.H., and Forsyth, L.A. (2003). *Motivating People to Be Physically Active.* Champaign, Ill.: Human Kinetics.

Miller, W.R., and Rollnick, S. (2004). *Motivierende Gesprächsführung.* Freiburg im Breisgau: Lambertus.

Nelson, M.E., Rejeski, W.J., Blair, S.N., Duncan, P.W., Judge, J.O., King, A.C., Macera, C.A., and Castaneda-Sceppa, C. (2007). Physical activity and public health in older adults: recommendation from the American College of Sports Medicine and the American Heart Association. *Medicine and Science in Sports and Exercise* 39**,** 1435-1445. doi: 10.1249/mss.0b013e3180616aa2.

Petrelli, A., Kaesberg, S., Barbe, M.T., Timmermann, L., Fink, G.R., Kessler, J., and Kalbe, E. (2014). Effects of cognitive training in Parkinson's Disease: a randomized controlled trial. *Parkinsonism & Related Disorders* 20**,** 1196-1202.

Petrelli, A., Kaesberg, S., Barbe, M.T., Timmermann, L., Rosen, J.B., Fink, G.R., Kessler, J., and Kalbe, E. (2015). One-year follow-up of cognitive training in Parkinson's disease. *European Journal of Neurology* 22**,** 640–647. doi: 10.1111/ene.12621.

Rahe, J., Liesk, J., Rosen, J.B., Petrelli, A., Kaesberg, S., Onur, O.A., Kessler, J., Fink, G.R., and Kalbe, E. (2015a). Sex differences in cognitive training effects of patients with amnestic mild cognitive impairment. *Aging, Neuropsychology, and Cognition***,** 1-19. doi: 10.1080/13825585.2015.1028883.

Rahe, J., Petrelli, A., Kaesberg, S., Fink, G.R., Kessler, J., and Kalbe, E. (2015b). Effects of cognitive training with additional physical activity compared to pure cognitive training in healthy older adults. *Clinical Interventions in Aging* 10**,** 297-310. doi: 10.2147/CIA.S74071.

Scarmeas, N., Stern, Y., Mayeux, R., Manly, J., Schupf, N., and Luchsinger, J.A. (2009). Mediterranean Diet and Mild Cognitive Impairment. *Archives of Neurology* 66**,** 216-225. doi: 10.1001/archneurol.2008.536.

Scarmeas, N., Stern, Y., Tang, M.-X., Mayeux, R., and Luchsinger, J.A. (2006). Mediterranean Diet and risk for Alzheimer's Disease. *Annals of Neurology* 59**,** 912-921. doi: 10.1002/ana.20854.
